# The extent of anticholinergic burden across an older Welsh population living with frailty: cross-sectional analysis of general practice records

**DOI:** 10.1093/ageing/afad136

**Published:** 2023-07-27

**Authors:** V-Lin Cheong, David Mehdizadeh, Oliver M Todd, Peter Gardner, Hadar Zaman, Andrew Clegg, David P Alldred, Muhammad Faisal

**Affiliations:** Medicines Management & Pharmacy Services, Leeds Teaching Hospitals NHS Trust, Leeds, UK; School of Healthcare, University of Leeds, Leeds, UK; School of Pharmacy and Medical Sciences, University of Bradford, Bradford, UK; NIHR Yorkshire and Humber Patient Safety Translational Research Centre, Bradford, UK; Academic Unit for Ageing and Stroke Research (University of Leeds), Bradford Institute for Health Research, Bradford Teaching Hospitals NHS Foundation Trust, Bradford, UK; School of Pharmacy and Medical Sciences, University of Bradford, Bradford, UK; NIHR Yorkshire and Humber Patient Safety Translational Research Centre, Bradford, UK; Wolfson Centre for Applied Health Research, Bradford, UK; School of Pharmacy and Medical Sciences, University of Bradford, Bradford, UK; NIHR Yorkshire and Humber Patient Safety Translational Research Centre, Bradford, UK; Wolfson Centre for Applied Health Research, Bradford, UK; NIHR Yorkshire and Humber Patient Safety Translational Research Centre, Bradford, UK; Academic Unit for Ageing and Stroke Research (University of Leeds), Bradford Institute for Health Research, Bradford Teaching Hospitals NHS Foundation Trust, Bradford, UK; Wolfson Centre for Applied Health Research, Bradford, UK; School of Healthcare, University of Leeds, Leeds, UK; NIHR Yorkshire and Humber Patient Safety Translational Research Centre, Bradford, UK; Wolfson Centre for Applied Health Research, Bradford, UK; NIHR Yorkshire and Humber Patient Safety Translational Research Centre, Bradford, UK; Wolfson Centre for Applied Health Research, Bradford, UK; Faculty of Health Studies, University of Bradford, Bradford, UK

**Keywords:** anticholinergic burden, frailty, older adults, routine data, structured medication review, older people

## Abstract

**Background:**

Anticholinergic medicines are associated with adverse outcomes for older people. However, little is known about their use in frailty. The objectives were to (i) investigate the prevalence of anticholinergic prescribing for older patients, and (ii) examine anticholinergic burden according to frailty status.

**Methods:**

Cross-sectional analysis of Welsh primary care data from the Secure Anonymised Information Linkage databank including patients aged ≥65 at their first GP consultation between 1 January and 31 December 2018. Frailty was identified using the electronic Frailty Index and anticholinergic burden using the Anticholinergic Cognitive Burden (ACB) scale. Descriptive analysis and logistic regression were conducted to (i) describe the type and frequency of anticholinergics prescribed; and (ii) to estimate the association between frailty and cumulative ACB score (ACB-Sum).

**Results:**

In this study of 529,095 patients, 47.4% of patients receiving any prescription medications were prescribed at least one anticholinergic medicine. Adjusted regression analysis showed that patients with increasing frailty had higher odds of having an ACB-Sum of >3 compared with patients who were fit (mild frailty, adj OR 1.062 (95%CI 1.061–1.064), moderate frailty, adj OR 1.134 (95%CI 1.131–1.136), severe frailty, adj OR 1.208 (95%CI 1.203–1.213)).

**Conclusions:**

Anticholinergic prescribing was high in this older population. Older people with advancing frailty are exposed to the highest anticholinergic burden despite being the most vulnerable to the associated adverse effects. Older people with advancing frailty should be considered for medicines review to prevent overaccumulation of anticholinergic medications, given the risks of functional and cognitive decline that frailty presents.

## Key Points

- The prevalence of prescribed anticholinergic medicines in older people living with frailty is under-investigated.
- We used routinely collected population-level patient data to investigate anticholinergic burden in older people stratified by frailty.
- Results demonstrated an association between severe frailty and higher anticholinergic burden.
- Frailty could be used to target structured medication review with a view to reduce or avoid anticholinergic medicines.

## Introduction

More than 30% of older people are estimated to be prescribed anticholinergic medications [1], which block the neurotransmitter acetylcholine in the central and peripheral nervous system. This can either be as an intended therapeutic effect, for example, in the treatment of conditions including chronic obstructive pulmonary disease, urinary incontinence and allergic rhinitis or as an unwanted side effect [2]. Importantly, the accumulation of multiple anticholinergic medicines in older populations is also associated with an increased risk of adverse outcomes including physical dysfunction, cognitive decline, hospitalisation and all-cause mortality [3, 4]. The cumulative effect of taking one or more anticholinergic medicine has been referred to as anticholinergic burden [5]. Despite concerns about adverse outcomes associated with anticholinergic prescribing in older people, the prevalence of anticholinergic prescribing in the UK continues to increase [6, 7]. This may be due to the burden of increasing multimorbidity within ageing populations, sub-optimal management with escalating polypharmacy, and the impact of the prescribing cascade whereby reported adverse effects of medicines are managed with the addition of further medicines [8, 9].

Given the projected increase in ageing across populations worldwide [10], anticholinergic burden is a global concern. Reduction of anticholinergic burden has been identified as a priority area within medicines optimisation in the UK, as is the reduction of polypharmacy [11]. Older people have greater susceptibility to experiencing adverse effects of medicines, and this is thought to be even more pronounced in the context of frailty—a condition characterised by a loss of biological reserves across multiple physiological systems [12–14].

As frailty is associated with vulnerability to adverse health outcomes [15], and a superior predictor of adverse outcomes compared with chronological age alone, risk stratification by frailty status may have utility in targeting medicines optimisation processes. The electronic Frailty Index (eFI) is now widely and freely available to help support the identification of frailty in UK primary care as it has been implemented in all UK suppliers of primary care electronic health record (EHR) systems [16]. The eFI has been extensively validated and demonstrates moderate to good agreement with research standard frailty measures [17].

In the UK, the NHS has introduced structured medication reviews (SMRs) as a contractual requirement for primary care networks (PCNs)—geographical groups of general practices working together to provide a wider range of services, covering populations of 30–50,000 patients. Targeting SMRs for older people with severe frailty as a potentially high-risk group (with identification supported using the eFI), and targeting based on anticholinergic burden for medicines review and rationalisation as an area of high-risk prescribing, are specified in the PCN contract. Although the targeting of frailty for medicines optimisation services is underway in primary care, few studies have investigated the impact of anticholinergic burden within populations of older people stratified by frailty severity. Furthermore, no studies have used routinely collected patient data at a population level to investigate anticholinergic burden in older people, stratified by frailty severity.

In this study, we aimed to investigate the prevalence of anticholinergic prescribing among older patients, describe the population by the frailty level and examine the anticholinergic burden in patients according to the level of frailty.

## Methods

### Study design, study setting and data

This was a cross-sectional analysis of primary care EHRs for the whole population of Wales using the Secure Anonymised Information Linkage (SAIL) databank [16]. The SAIL databank is a national data safe haven of de-identified datasets from the population of Wales, which includes records of over 5 million people who have been recipients of public services [18]. The inclusion criteria for this study were: all registered patients who were alive and aged 65 or older at their first GP consultation between 1 January and 31 December 2018; patients permanently registered with a medical practice for a minimum of 12 months prior to the consultation. No exclusion criteria were applied. The study investigators only had access to the database population as per the criteria specified above to create the study population, and therefore did not have access to the entire population of Wales within the SAIL databank.

### Measurement of anticholinergic burden

All medicines prescribed to each patient were classified according to an updated and adapted Anticholinergic Cognitive Burden (ACB) scale [19, 20]. The final ACB scale that was used had been specifically adapted to medicines available in the UK in a study also using primary care EHR data [20]. The ACB scale uses a scoring system of 0 to 3, with 0 indicating unlikely anticholinergic activity, 1 indicating possible, and 2 and 3 indicating those with definite anticholinergic activity [19]. The total cumulative score of a patient taking one or more medications with anticholinergic activity was then quantified as a sum of the scores for the individual medications (ACB-Sum). A master sheet containing all relevant anticholinergic medicines in the updated ACB scale was developed and mapped to the relevant Read version 2 code, which is the primary care coding terminology used in SAIL, so that the medicines could be characterised as per the ACB scale. The ACB-Sum score for each patient was calculated by identifying every anticholinergic medicine prescribed over a 28-day period prior to the first GP consultation in year 2018, regardless of whether it was issued as a repeat or acute prescription. Duplicate and locally acting medicines (e.g. creams, nebulisers, eye drops, etc.) were removed to avoid overestimation of the ACB-Sum score.

Anticholinergic medicines were then categorised into drug class in accordance with British National Formulary classification, and Anatomical Therapeutic Chemical (ATC) classifications.

### Measurement of frailty

Frailty was measured using the eFI which was developed and validated in primary care EHR data sets [16]. The eFI is based on the internationally recognised cumulative deficit model [21], which identifies frailty on the basis of the accumulation of a range of health deficits spanning clinical signs, symptoms, diseases, disabilities, impairments and abnormal laboratory data [22]. An eFI score between 0 and 1 is automatically calculated based on the identified presence of any of 36 equally weighted variables in the primary care EHR data. Where frailty is categorised in the analysis, patients with an eFI score < 0.12 are identified as fit; ≥0.12 and <0.24 as having mild frailty; ≥0.24 and <0.36 as having moderate frailty; and ≥0.36 as having severe frailty [16].

### Co-variables

A number of covariables were defined and characterised from the dataset, including deprivation, ethnicity and living circumstances. Deprivation of the cohort was derived and categorised using Welsh Index of Multiple Deprivation (WIMD) [23]. Ethnicity data was derived from NHS ethnicity codes on the SAIL database. Lastly, living circumstances for this study was categorised by housebound status, derived from whether a ‘housebound’ code was recorded.

### Statistical analyses

Descriptive analysis was conducted to summarise the study population according to key demographics including age, sex, living circumstances and comorbidities. Sub-groups of older people defined by frailty category were compared on these parameters. Each parameter was checked for its distribution, whether normal or non-normal to determine whether respective parametric or non-parametric summary measures should be applied.

The population was summarised according to:

- prevalence of any anticholinergic medication use; defined as at least one anticholinergic medicine;
- average ACB-Sum score;
- proportion of patients on medicines with an ACB score of 3 (regarded as the most potent anticholinergics);
- prevalence of any anticholinergic medication use, by class.

A logistic regression model was used to estimate the association between the frailty categories (fit, mild frailty, moderate frailty, severe frailty) as the exposure, and ACB- Sum score (high/low with ACB-Sum >3 as high and < 3 as low) as the outcome, with adjustment for relevant covariates (sex, age, living circumstances, deprivation and ethnicity). We calculated unadjusted and adjusted Odds Ratios (OR) including 95% confidence intervals (CI). We followed the Reporting of studies Conducted using Observational Routinely-collected Data (RECORD) guidelines [24] for reporting the results of this study (Supplementary Table 3), as an extension of the Strengthening the Reporting of Observational studies in Epidemiology (STROBE) guidelines.

### Ethical approval

This secondary analysis of routinely collected patient data was conducted in accordance with section 254 of the UK Health and Social Care Act 2012. The data used in this study are available in the SAIL Databank at Swansea University, Swansea, UK. Permission to use SAIL data was sought, reviewed and approved by the SAIL Information Governance Review Panel (IGRP ref 0978).

## Results

**Figure 1:**
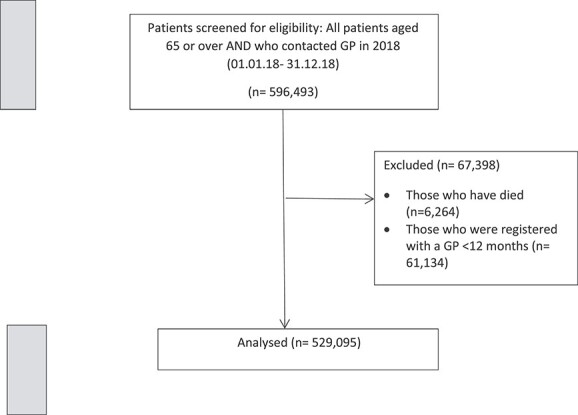
STROBE flowchart of cohort selection.

The study population included 529,095 patients. The derivation of the analytic cohort is outlined in Figure 1. The mean age was 75.0 years (SD = 7.4) and 284,810 were women (53.8%) (Table 1). In terms of ethnicity, 45.5% were white, <0.1% were non-white, and 54.5% had no ethnicity stated or had missing data. In relation to deprivation, 15.5% of the overall cohort were in the most deprived WIMD quintile, and the prevalence of those in the most deprived quintile increased with frailty severity. The population cohort was stratified by frailty status as follows: 255,402 people (48.3%) as fit; 185,902 with mild frailty (35.1%); 69,867 with moderate frailty (13.2%); and 17,924 with severe frailty (3.4%). The mean age increased with increasing frailty: from 72.5 years (SD = 6.1) in the fit group, to 82.4 years (SD = 7.6) in the severe frailty group. The proportion of people who were housebound also increased with increasing frailty: from 4.7% in the fit group to 69.8% in the severe frailty group. Comorbidity prevalence also increased with increasing frailty (Supplementary Table 1).

**Table 1:** Characteristics of SAIL cohort patients as a whole and by their electronic frailty index (eFI) groups

Characteristic	All *n* = 529,095	Fit *n* = 255,402	Mild frailty *n* = 185,902	Moderate frailty *n* = 69,867	Severe frailty *n* = 17,924
Mean age [years] (SD)	75.0 (7.4)	72.5 (6.1)	76.0 (7.3)	79.6 (7.9)	82.4 (7.6)
Sex [male] (%)	244,285 (46.2)	129,144 (50.6)	81,667 (43.9)	27,217 (39.0)	6,257 (34.9)
Housebound (%)	95,671 (18.1)	11,922 (4.7)	39,217 (21.1)	32,023 (45.8)	12,509 (69.8)
Ethnicity					
White (%)	240,476 (45.5)	126,290 (49.4)	88,367 (47.5)	22,301 (31.9)	3,518 (19.6)
Asian (%)	120 (<0.1)	59 (<0.1)	43 (<0.1)	14 (<0.1)	<10 (<0.1)
Black (%)	124 (<0.1)	81 (<0.1)	34 (<0.1)	<10 (<0.1)	<10 (<0.1)
Mixed (%)	115 (<0.1)	62 (<0.1)	44 (<0.1)	<10 (<0.1)	<10 (<0.1)
Missing (%)	288,260 (54.5)	128,910 (50.5)	97,414 (52.4)	47,534 (68.0)	14,402 (80.4)
WIMD					
1 (most deprived) (%)	82,064 (15.5)	34,268 (13.4)	31,429 (16.9)	12,994 (19.6)	3,373 (18.8)
2 (%)	101,734 (19.2)	45,717 (17.9)	37,615 (20.2)	14,575 (20.9)	3,827 (21.4)
3 (%)	110,871 (21.0)	53,049 (20.7)	38,957 (21.0)	14,947 (21.4)	3,918 (21.9)
4 (%)	106,969 (20.2)	53,456 (20.9)	36,409 (19.6)	13,502 (19.3)	3,602 (20.0)
5 (least deprived) (%)	120,805 (22.8)	65,238 (25.5)	39,410 (21.2)	13,124 (18.8)	3,033 (16.7)
Missing (%)	6,652 (1.3)	3,674 (1.4)	2082 (1.1)	725 (1.0)	171 (0.9)
Median index of deprivation (IQR)	3.0 (2.0)	3.0 (3.0)	3.0 (2.0)	3.0 (2.0)	3.0 (3.0)
Prescribed at least one anticholinergic medicine (%)	250,850 (47.4)	78,515 (30.7)	107,317 (57.7)	50,495 (72.3)	14,523 (81.0)
Mean number of ACB medicines prescribed (SD)	0.8 (1.1)	0.4 (0.7)	1.0 (1.1)	1.4 (1.3)	1.8 (1.4)
Prevalence of prescribed medicine with:					
ACB score 1 (%)	233,217 (44.1)	72,010 (28.2)	99,685 (53.6)	47,608 (68.1)	13,914 (77.6)
ACB score 2 (%)	669 (0.1)	170 (<0.1)	269 (0.2)	168 (0.2)	62 (0.4)
ACB score 3 (%)	48,767 (9.2)	12,508 (4.9)	21,582 (11.6)	11,239 (16.1)	3,438 (19.2)
Mean ACB-Sum score (SD)	1.0 (1.5)	0.5 (1.0)	1.2 (1.5)	1.8 (1.8)	2.2 (1.9)
ACB-Sum score < =3	490,421 (92.7)	248,486 (97.3)	169,091 (91.0)	58,923 (84.3)	13,921 (77.7)
ACB-Sum score > 3 and < =6	34,532 (6.5)	6,437 (2.5)	15,160 (8.2)	9,547 (13.7)	3,388 (18.9)
ACB-Sum score > 6 and < =9	3,827 (0.7)	448 (0.2)	1,548 (0.8)	1,264 (1.8)	567 (3.2)
ACB-Sum score > 9	315 (<0.1)	31 (<0.1)	103 (<0.1)	133 (0.2)	48 (0.3)

ACB, Anticholinergic Cognitive Burden; ACB-Sum, Sum of the Anticholinergic Cognitive Burden score; IQR, interquartile range; SD, standard deviation; WIMD, Welsh Index of Multiple Deprivation

### Prevalence of any anticholinergic medicine use

At least one anticholinergic medicine was prescribed to 47.4% of the population cohort (Table 1). An examination of the frailty severity subgroups found that 30.7% of ‘fit’ patients were prescribed an anticholinergic medicine, increasing to 57.7% in ‘mildly frail’ patients, 72.3% of ‘moderately frail’ patients and 81.0% of ‘severely frail’ patients. Similarly, the mean number of anticholinergic medicines also increased with increasing frailty.

### Average ACB-Sum score

**Figure 2:**
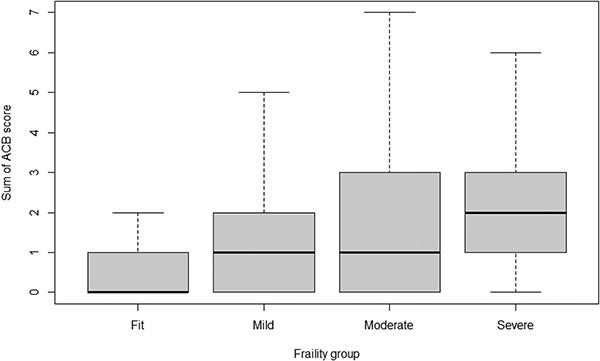
Relationship of Sum of Anticholinergic Cognitive Burden (ACB) (ACB-Sum) score with eFI groups.

The overall mean ACB-Sum for the population cohort was 1.0 (SD = 1.5), with the mean ACB-Sum ranging from 0.5 for fit patients to 2.2 for patients with severe frailty, increasing with increasing frailty (Figure 2). Importantly, the number of patients having an ACB-Sum of >3 and ≤ 6 also increased, from 8.2% in those with mild frailty, to 18.9% in those with severe frailty. A similar trend of increasing proportions of patients with an increased ACB-Sum with increasing frailty was also observed in those with ACB-Sum of >6 to ≤9 and ACB-Sum of >9.

### Proportion of patients on potent anticholinergic medicines (ACB score of 3)

The prevalence of prescribed medicines with an ACB score of 3 for the overall population was 9.2%. The prevalence of prescribed medicines with an ACB score of 3 was higher in those with severe frailty compared with those with mild frailty (19.2% vs. 16.1%). This trend was also consistent across medicines with an ACB score of 1 and 2.

### Anticholinergic medicines by drug class

The 25 most frequently prescribed medications with anticholinergic activity are presented in Supplementary Table 2 in a descending order, stratified by frailty severity. A large proportion of the anticholinergic medicines prescribed had an ACB score of 1. The two most frequently prescribed medication groups with an ACB score of 3 in the study population were antidepressants (4.7%) and medications for urinary incontinence (1.3%). The three most frequently prescribed medications with ACB score of 3 were amitriptyline (4.7%) (tricyclic antidepressant), solifenacin (1.3%) (antimuscarinic used for bladder instability) and paroxetine (0.5%) [selective seronotinin reuptake inhibitor (SSRI)]. Amitriptyline was frequently prescribed in all the frailty groups.

There were three medications with an ACB score of 2 prescribed for patients: carbamazepine (antiepileptic), amantadine (anti-Parkinson’s) and nefopam (analgesic), prescribed for <1% of the total cohort of patients.

Prescribed medications with an ACB score of 1 belonged to a diverse range of ATC classifications. Overall, the most frequently prescribed were diuretics (17.9%), followed by antidepressants (7.5%), and antihypertensives (7.4%). The specific medications prescribed under these medication classes were as follows: thiazide and loop diuretics (bendroflumethiazide, indapamide, furosemide), antihypertensives (atenolol, doxazosin), antidepressants (citalopram, fluoxetine, mirtazapine).

In general, there was a pattern of increasing proportion of patients prescribed anticholinergic medicines with increasing frailty. For example, citalopram, an SSRI, was prescribed for 2.3% of those in the fit category, 4.6% in the mild frailty category, 7.6% in the ‘severe frailty’ category. A similar pattern was observed for amitriptyline, solifenacin, paroxetine and oxybutynin with anticholinergic burden scores of 3.

### Regression analysis

Patients with increasing frailty had higher odds of having an ACB-Sum of >3 compared with patients who were fit [mild frailty, adj OR 1.062 (95%CI 1.061–1.064), moderate frailty, adj OR 1.134 (95%CI 1.131–1.136), severe frailty, adj OR 1.208 (95%CI 1.203–1.213)] (Table 2).

**Table 2:** Regression analysis of ACB-Sum>3 vs frailty status

ACB-Sum > 3 as a binary outcome	Crude OR (95%CI)	^a^Adjusted OR (95%CI)
Fit (Reference)	—	—
Mild frailty	1.065 (1.064 to 1.067)	1.062 (1.061 to 1.064)
Moderate frailty	1.138 (1.136 to 1.141)	1.134 (1.131 to 1.136)
Severe frailty	1.217 (1.212 to 1.222)	1.208 (1.203 to 1.213)

^a^Adjusted for Age, Sex, Housebound, Ethnicity, Welsh Index of Multiple Deprivation (WIMD)

## Discussion

In this study of 529,095 patients aged 65 years or older, it was found that 47.4% of all study participants, and 81% of patients identified with severe frailty using the eFI, received at least one anticholinergic medicine. The medication classes most frequently prescribed in the population overall were antidepressants, medications for urinary diseases, diuretics and antihypertensives.

Our study found that people with severe frailty were prescribed a higher number of medicines with anticholinergic properties and had a higher cumulative ACB-Sum compared with those living without frailty and those living with mild or moderate frailty. Given that anticholinergics can have a significant impact on morbidity and mortality [20], the increased anticholinergic burden in patients living with frailty is concerning. This is especially so because the severity of frailty is also associated with increased risk of adverse drug reactions and mortality [16]. Despite the general consensus that anticholinergic exposure should be limited in older people where possible, our study showed that they are commonly prescribed, especially in patients with severe frailty. A number of previous studies have reported on the prescribing rate of at least one medicine with ACB properties. For instance, a study conducted in the general practice population in Slovenia found the prevalence of anticholinergic prescribing to be 12.5% [25], which was lower than the rate of prescribing found in our study (47.4%). However, there was a difference in the tools used to calculate anticholinergic burden between the study by Gorup and colleagues [25] and ours, where the former used a combined ACB calculation of Duran scale [26] and DBI-ACH score [27] which took into account of dosages, whereas the ACB score used in this study did not. Furthermore, in the UK, Grossi et al. [6] examined and compared the prevalence of medicines with ACB properties across two time periods: 1990/1993 and 2008/2011 and found an increase from 49.6% in 1990/1993 to 64.3% in 2008/2011. The higher prevalence of medicines with ACB properties reported by Grossi and colleagues could be at least partially attributed to the difference in methods and medicines lists used to calculate ACB score compared with ours [6]. Similarly, the authors also found a statistically significant increase in overall prevalence of potent anticholinergic use among the over 65 s (from 5.7% in 1990/1993 to 9.9% in 2008/2011).

To our knowledge, this is the first study to have examined the prevalence of anticholinergic burden in people with different levels of frailty severity using the eFI. More importantly, this study is the first study to find an association between increased ACB-Sum and increasing severity of frailty. It is possible that the increased prevalence of anticholinergic prescribing in patients living with frailty may be explained by the association between the method used to identify frailty and multimorbidity. As the eFI adopts the cumulative deficit model for frailty characterisation, patients with a greater number of deficits are likely to have co-morbidities and clinical features that can subsequently lead to an increase in the use of multiple anticholinergic medicines to manage such co-morbidities. Nevertheless, a high-risk population who are least likely to tolerate the adverse effects associated with anticholinergic burden, yet exposed to the highest levels of anticholinergic burden, is a paradox that is of potential clinical concern. Due to the increased vulnerability to adverse effects of anticholinergic burden in frailty, there is a strong case for using safer alternatives to prevent overaccumulation of anticholinergics, or rationalising anticholinergic medicines in frailty, where possible. We acknowledge that in many instances, anticholinergic medicines are indicated for the frail to manage long term conditions, such as cardiac conditions as an example, or potent anticholinergics to treat bladder instability. Therefore, the use of medicines with anticholinergic activity does not necessarily indicate a suboptimal prescription. However, although it is important to consider the appropriateness of individual anticholinergic medicines, it is also important to consider their impact collectively in the context of anticholinergic burden, with evidence demonstrating increased associations with adverse events with greater cumulative exposure. We recommend that patients should be prioritised for review based on frailty severity, and anticholinergic burden scores, and therapies optimised to reduce overall anticholinergic burden where possible and clinically appropriate. In general, our findings provide support for the NHS England SMR contract, by confirming that targeting of SMRs based on frailty status is more likely to identify older people at an increased risk of adverse outcomes who are also more likely to be prescribed potentially harmful anticholinergic medications.

The association identified in this study between frailty as measured by the eFI and anticholinergic prescribing presents an opportunity in clinical practice. On the basis of these findings, the eFI could be used as a way to identify people at a risk of high anticholinergic burden to effectively target the delivery of anticholinergic burden reduction strategies. More importantly, given the effects of both anticholinergic burden and frailty in increasing morbidity and mortality, there is also an opportunity to use a combination of ACB and the eFI, as that implemented in the AC-frail tool [13], or the ACMI tool [28] to allow proactive prioritisation of patients for the delivery of SMRs. A recent Cochrane review however, emphasised the uncertainty in evidence around the reversibility of adverse effects upon cessation of anticholinergics [29]. There might therefore, also be an opportunity to create reminders and alerts for patients with frailty to avoid the initiation of anticholinergics, and unnecessary overaccumulation where possible.

### Strengths and limitations

The key strengths of this study were that the analyses are based on a large sample (over 500,000 people aged 65 and older) taken from a nationwide population; and that the methods used to categorise both ACB and frailty are well founded. Frailty was measured using a method accessible to the majority of general practitioners in the UK, hence improving the clinical practice and policy relevance of the study findings.

In common with existing measures of anticholinergic burden, the ACB score does not include adjustment for dosage, duration of prescriptions or adherence which means that there are uncertainties regarding the impact of cumulative dose of medications for older people with frailty. The cross-sectional design does not allow for prospective investigation of the association between anticholinergic medications, frailty and outcomes, or examine anticholinergic medication prescribing trajectories over time which would be necessary to address questions about causal inference.

## Conclusion

Anticholinergic prescribing was high in this older population and was more prevalent in older people living with advancing frailty compared with those without frailty. Clinical manifestations associated with worsening frailty (defined by the eFI) may well explain the relationship with greater anticholinergic prescribing, and why higher ACB-Sum scores may be expected. Overaccumulation of anticholinergic medications is a concern for people with advancing frailty as they are less likely to tolerate the associated adverse effects. Given the risks of functional and cognitive decline that living with frailty presents, careful review of anticholinergic medications is needed to ensure therapies are optimised, and rationalised if they are no longer in the best interest of patients. Patients living with frailty should be a high priority for interventions such as SMRs, to reduce anticholinergic burden where appropriate.

## Supplementary Material

aa-22-1490-File002_afad136

## Data Availability

The data used for the study is third-party data and is held by the SAIL Databank at Swansea University on behalf of health care providers in Wales who are the original data owners. This study was approved by the Secure Anonymised Information Linkage (SAIL) Information Governance Review Panel (IGRP) (project 0978) in Wales. All data were anonymised prior to access and analysis. We did not have special access to this data; it is available to anyone via an application to SAIL. All proposals to use SAIL data are subject to review by an independent Information Governance Review Panel (IGRP). Before any data can be accessed, approval must be given by the IGRP. The IGRP gives careful consideration to each project to ensure proper and appropriate use of SAIL data. When access has been approved, it is gained through a privacy protecting safe haven and remote access system referred to as the SAIL Gateway. SAIL has established an application process to be followed by anyone who would like to access data via SAIL https://www.saildatabank.com/application-process.
